# Evaluation of preservation methods for improving biogas production and enzymatic conversion yields of annual crops

**DOI:** 10.1186/1754-6834-4-20

**Published:** 2011-07-19

**Authors:** Annukka Pakarinen, Pekka Maijala, Seija Jaakkola, Frederick L Stoddard, Maritta Kymäläinen, Liisa Viikari

**Affiliations:** 1 University of Helsinki, Department of Food and Environmental Sciences, PO Box 27, 00014 Helsinki, Finland; 2 University of Helsinki, Department of Agricultural Sciences, PO Box 27, 00014 Helsinki, Finland; 3 HAMK University of Applied Sciences, PO BOX 230, 13101 Hämeenlinna, Finland

## Abstract

**Background:**

The use of energy crops and agricultural residues is expected to increase to fulfil the legislative demands of bio-based components in transport fuels. Ensiling methods, adapted from the feed sector, are suitable storage methods to preserve fresh crops throughout the year for, for example, biogas production. Various preservation methods, namely ensiling with and without acid addition for whole crop maize, fibre hemp and faba bean were investigated. For the drier fibre hemp, alkaline urea treatment was studied as well. These treatments were also explored as mild pretreatment methods to improve the disassembly and hydrolysis of these lignocellulosic substrates.

**Results:**

The investigated storage treatments increased the availability of the substrates for biogas production from hemp and in most cases from whole maize but not from faba bean. Ensiling of hemp, without or with addition of formic acid, increased methane production by more than 50% compared to fresh hemp. Ensiling resulted in substantially increased methane yields also from maize, and the use of formic acid in ensiling of maize further enhanced methane yields by 16%, as compared with fresh maize. Ensiled faba bean, in contrast, yielded somewhat less methane than the fresh material. Acidic additives preserved and even increased the amount of the valuable water-soluble carbohydrates during storage, which affected most significantly the enzymatic hydrolysis yield of maize. However, preservation without additives decreased the enzymatic hydrolysis yield especially in maize, due to its high content of soluble sugars that were already converted to acids during storage. Urea-based preservation significantly increased the enzymatic hydrolysability of hemp. Hemp, preserved with urea, produced the highest carbohydrate increase of 46% in enzymatic hydrolysis as compared to the fresh material. Alkaline pretreatment conditions of hemp improved also the methane yields.

**Conclusions:**

The results of the present work show that ensiling and alkaline preservation of fresh crop materials are useful pretreatment methods for methane production. Improvements in enzymatic hydrolysis were also promising. While all three crops still require a more powerful pretreatment to release the maximum amount of carbohydrates, anaerobic preservation is clearly a suitable storage and pretreatment method prior to production of platform sugars from fresh crops.

## Background

Ensiling of fresh crops is a necessary and common procedure to provide nutrient-rich fodder for animals throughout the year, especially where the growing period is short. The increasing use of energy crops and residues needed as raw materials to fulfil the requirements of bio-based transport fuels can also benefit from preservation of the raw material. Energy and yield savings in the conversion process of the raw material can be obtained through efficient storage methods that avoid the expense of drying, while still preserving the valuable carbohydrates. Furthermore, ensiling may function as a beneficial pretreatment for lignocellulosic materials before further processing to methane or ethanol. Ensiling is normally carried out in anaerobic conditions in which organic acids, especially lactic and acetic acids produced by endogenous microflora, decrease the pH, preserve the substrate against growth of fungi, bacteria or yeasts, and thus prevent carbohydrate losses. During the fermentation, acids are formed from the easily available non-structural carbohydrates, as well as from structural carbohydrates that become available during prolonged storage as a result of hydrolysis by acids and endogenous enzymes [1,2].

Acids, especially formic acid in the Nordic countries, are commonly added to initiate the preservation [3]. Requirements to increase the nutritional value of animal feed and to preserve the carbohydrates during the storage have led to increased research on, for example, urea and acidic additives [4,5]. Acidic pretreatments have been found to enhance the production of biogas or bioethanol by decreasing the crystallinity of the cellulosic material [6], by increasing the accessible surface area of plant substrates, and by altering lignin structure [7]. Dilute sulfuric or hydrochloric acids, as well as organic acids, are commonly used for pretreatment of various raw materials prior to enzymatic hydrolysis [8], but usually in significantly more severe conditions than those applicable for ensiling. Sulfuric acid treatment prior to anaerobic ensiling was found to enhance the conversion of reed canary grass and switch grass to ethanol [9], indicating that lower levels of acid amendments to ensilage can have the desired effects.

Alkaline conditions may also be used for storing crops. Urea treatments, especially on late-harvested crops, were found to preserve the carbohydrate and nutrient contents during storage [4] and the added urea improved slightly the nutritive value of nitrogen-poor crops, such as whole-crop maize, as feed for ruminants [10]. Alkaline preservation has been found to improve also the enzymatic conversion [9,11-13]. Treatments with sodium hydroxide or urea in various conditions have been observed to cause swelling, to decrease the degree of polymerisation and crystallinity of cellulose, and expectedly, to degrade linkages between lignin and carbohydrates [14].

Decomposition of cell walls during ensiling could improve the availability of nutrients and carbohydrates for the methanogens and thus enhance methane production [15]. Ensiled corn stover and grasses are commonly used raw materials in present methane production plants. Due to the increased formation of lactic and acetic acids in ensiling, higher methane yields have been obtained [15,16]. Formic acid, used as an additive in ensiling, preserved the grass substrate well and enhanced methane yields [3], whereas biological additives, such as lactic acid bacteria or hydrolytic enzymes, had inconsistent effects on methane yields, mainly because of suboptimal ensiling methods [16-18]. However, addition of cellulolytic and hemicellulolytic enzymes to ensiling increased the amount of water-soluble carbohydrates and formation of lactic acid and was suggested as an effective pretreatment for the fibrous kenaf [19]. Minimal carbohydrate losses during storage are essential for economic production of ethanol from energy crops. Ensiling of lignocellulosic materials has been found to be a promising and cost-effective storage and pretreatment method for ethanol production [20,21], but the chemical modifications affecting the hydrolysability of ensiled crops have not been thoroughly examined. There is little published data on the use of urea as a pretreatment and storage agent of fresh crops prior to enzymatic conversion to sugars and further to fuels. Although ensiling is known to improve methane yields of many commonly used crops, the efficiency depends on plant species. In this work, three crops, maize, hemp and faba bean, producing high yields in boreal conditions [22], were ensiled without additives, as well as preserved with the addition of formic acid and urea. These three crops differ from each other in their chemical characteristics, hemp being rich in cellulose, lignin and pectin, maize in hemicelluloses, particularly xylans, and faba bean in starch and nitrogen [22]. While maize and faba bean are common raw materials used for ensiling for feed [23], ensiling of hemp is a novel approach, supported by its established status as a high yielding energy crop. In this work, detailed analysis of the chemical composition of the high yield crops after various storage methods and conditions was carried out, and the potential for methane production and enzymatic conversion of these raw materials was evaluated.

## Materials and methods

### Plant materials and preparation of materials for analyses

Three herbaceous crops, forage maize (*Zea mays*, cv. Ronaldino), fibre hemp (*Cannabis sativa*, cv. Uso) and faba bean (*Vicia faba*, cv. Aurora) were harvested from plots in Southern Finland in September 2008, and the same cultivar of hemp was also used in 2009. After cutting in 2008 maize was prewilted for 48 h and faba bean for 20 h to reduce the moisture content of the materials. No prewilting was necessary for hemp in 2008 while in 2009 hemp was wilted for 48 h. Crops were cut by a garden chopper into 1-2 cm size pieces and ensiled. Samples of fresh material were frozen at -22°C for later analysis.

For enzymatic hydrolysis, the raw material was milled with an IKA M20 universal mill (IKA^®^-Werke GmbH & Co. KG, Staufen, Germany), resulting in a maximum particle size of 7 mm. For chemical analyses, hemp and maize were freeze dried, whereas faba bean was dried at 60°C for 3 days. All dried samples were milled with an IKA A10 basic analytical grinder mill (IKA^®^-Werke GmbH & Co. KG, Staufen, Germany) to a maximum particle size of 1 mm. Prior to analysis, the dried and milled samples were extracted with acetone in an automatic Soxtec 2050 extractor (FOSS Analytical, Hilleroed, Denmark) for 0.5 h.

### Preservation

Laboratory-scale ensiling was conducted using 1.5 l glass jars (Weck; Wher-Oflingen, Wher, Germany) in three replicates. In 2008, chopped maize, hemp and faba bean were ensiled either without or with two dosages of formic acid, 0.5% w/w and 1% w/w of the fresh material, resulting in final concentrations of 1.7% and 3.4% (hemp), 3.0% and 6.0% (maize) and 2.1% and 4.2% (faba bean) of the dry matter (DM). The material was pressed tightly into the jars and sealed airtight. The density of the ensiled material simulated well full-scale ensiling systems, being 145-160 kg DM/m^3 ^for the maize, hemp and faba bean in 2008 and 250 kg DM/m^3 ^for the drier hemp in 2009. The jars were stored at 10°C and after 2 months the temperature was decreased to 5°C. In 2009, prewilted hemp was treated with two dosages of urea-water solution at 3% w/w and 6% w/w of the dry matter. An equivalent amount of water was added to control samples. The jars were stored at 10°C for the first month and at 5°C for the rest of the storage. Jar samples were withdrawn for testing after 4 and 8 months of storage. After the preservation period, the pH of each jar was measured, the material was visually examined and the replicates were combined for further use and chemical analyses. The urea treatment was investigated only for hemp, the only raw material that had a high enough dry matter content to be suitable for alkali preservation [24].

### Analyses of the fresh and ensiled materials

The dry matter (DM) content (or total solids TS%) was determined by drying the samples at 105°C until a constant weight was reached. The dried samples were combusted in a muffle oven for 2 h at 550°C to determine the ash content. The level of volatile solids (VS%) was calculated by subtracting the ash content from the dry matter content.

Lignin and carbohydrates were analysed according to the National Renewable Energy Laboratory Laboratory Analytical Procedure (NREL LAP) methods (Determination of Structural Carbohydrates and Lignin) [25]. In this method, acid hydrolysis is used to hydrolyse the lignocellulosic material into monosaccharides. Acid hydrolysis was performed in three replicates. Klason lignin was determined gravimetrically as the acid-insoluble residue from acid hydrolysis. Acid-insoluble ash was also present in the lignin residue. The protein was analysed from the samples but due to an unknown amount of acid insoluble protein it was not subtracted from the total acid insoluble residue after acid hydrolysis. Acid soluble lignin was determined from the filtrate from the acid hydrolysis spectrophotometrically at 320 nm. The amount of total sugars formed after acid and enzymatic hydrolysis was determined as reducing sugars by the dinitrosalicylic acid (DNS) method [26] at 540 nm. Reducing sugars were analysed from all three replicates of the acid hydrolysates and standard errors calculated from the results. One of the replicates (the middle one) was chosen for further monosaccharide analyses. The high performance anion exchange chromatography with pulsed amperometric detection (HPAEC-PAD) system was equipped with two Waters 515 HPLC pumps, a Waters 2707 autosampler, and Waters 2465 electrochemical detector using Empower software (Waters Corporation, Milford, MA, USA) for instrument control and data handling. The analytical CarboPac PA-1 with the guard column (Dionex Corporation, Sunnyvale, CA) was maintained at 30°C. For monosaccharides (glucose, xylose, mannose, arabinose, galactose and fructose) the eluents used for gradient analysis were (a) H_2_O and (b) 200 mM NaOH using the flow rate of 1 ml/min. The easily depolymerisable carbohydrates were analysed by acid methanolysis [27] in which the depolymerised carbohydrates were silylated and non-cellulosic glucose and uronic acids were determined by gas chromatography, Agilent 6890N (Agilent Technology, Palo Alto, CA, USA) equipped with FID detector. The column used was DB-1 (30 m × 0.32 mm × 0.25 mm). The conditions were as follows: oven temperature 150°C (5 min), 2°C/min to 186°C, followed by 1°C/min to 200°C and 20°C/min to 325°C, injector temperature was 225°C and FID temperature 280°C. The injection volume was 2 μL with a split ratio of 1:30 and the flow rate of the carrier gas helium was 1 ml/min. Cellulose content was calculated by subtracting the glucose fraction determined in acid methanolysis from the total glucose obtained in acid hydrolysis.

Water-soluble, readily available carbohydrates (WSC) were determined from starting samples of the enzymatic hydrolysis. Total reducing carbohydrates were analysed by the DNS method spectrophotometrically (at 540 nm) and monosaccharides with HPAEC-PAD. Organic acids (acetic, lactic, malic, formic and oxalic) in the filtrate pressed from the solid blended materials were analysed with Agilent 1100 series liquid chromatograph, equipped with a UV-VIS diode array detector, using external standards of each of the acids. The detection wavelength was 210 nm and the reference wavelength was 550 nm. Volatile fatty acids (propionic, butyric, valeric and isovaleric acids) were determined from the same supernatant with a Shimadzu GC-17-A- gas chromatograph with flame ionization detector (FID). The column used was Phenomex Zebron capillary GC column, phase: ZB-FFAP (30 m × 0.53 mm; 1 μm (df)). Temperatures were 70°C, 250°C, and 300°C for the column, injector and detector, respectively. The carrier gas used was helium and external standards were used of each of the acids

The total nitrogen and carbon contents of crops were determined by the Dumas combustion method using a Vario Max CN analyser (Elementar Analysensysteme GmbH, Hanau, Germany). Protein was calculated from the nitrogen content by multiplying by 6.25.

### Methane production

Methane production was determined in laboratory-scale batch trials. Each crop was treated in 100 ml bottles using eight replicates. Digested sludge from a municipal waste water treatment plant (Hämeenlinna, Finland) was used as an inoculum. The average pH of the inoculum was 7.9, the VS was 1.7% and the TS 3.6%. The VS ratio of sample and inoculum in each bottle was 1:1. The bottles were filled to 60 ml with distilled water and NaHCO_3 _(3 g/l) was added as buffer. The bottles were flushed with N_2_, closed, incubated in a water bath at 36 ± 1°C and mixed manually on a daily basis. The formed biogas was led into 1% NaOH solution to remove CO_2_. The volume of methane was determined by displacement of sodium hydroxide solution, daily at the beginning and less frequently as biogas production decreased. All methane production trials were carried out for 30 ± 2 days. For reference purposes, the methane yield produced by the inoculum alone was determined and subtracted from the sample yields. Methane yields were expressed as volume of methane Ndm^3^/kg (normal litre per kg) per corrected unit mass of VS. The ensiled materials contained more volatile compounds (acids, ammonium-N) than the raw materials. When the dry matter or volatile solids are determined by drying, the volatile compounds are partially lost. A correction factor for the dry matter is commonly used in biogas research [28]. The dry matter and volatile solids used for calculating the acid concentrations and biogas yields were corrected according to Huida *et al*. [29] with the following equation:

where ODM = oven dry matter, E = Ethanol, FA = formic acid, AA = acetic acid, PA = propionic acid, BA = butyric acid, IVA = isovaleric and valeric acid, LA = lactic acid and AN = ammonium nitrogen.

### Enzymatic hydrolysis

The accessibility of the crops for enzymatic conversion was studied by hydrolysing the raw materials with commercial enzyme mixtures. The basic cellulase mixture of Celluclast (Novozymes, Denmark) containing the major cellulolytic activities (dosage 10 filter paper units (FPU)/g DM biomass), was supplemented with the beta-glucosidase preparation, Novozym 188 (500 nanokatals (nkat)/g DM biomass). The dry matter content of the milled crop in the hydrolysis was 2%. The experiments, with two replicates, were carried out in a test tube shaker in 5 ml in 50 mM Na-citrate buffer, pH 5, and 0.02% of NaN_3 _was added to prevent bacterial growth during the hydrolysis. The temperature was 50°C and the shaking speed 200 rpm. Samples were taken at the beginning of experiments and after 48 h of incubation. Reducing sugars and monosaccharides were determined from the hydrolysates as described above.

### Statistical evaluation

Changes in chemical composition, and effect on ensiling for methane yields and enzymatic hydrolysability were tested with the t test using PASW version 18.0 (SPSS Inc., Chicago, IL, USA). Statistical significance was recognised for p < 0.05.

## Results

### Chemical composition of crops

The fresh crops showed distinct differences in their chemical composition. The cellulose and lignin contents were highest in hemp, 37% and 17%, intermediate at 23% and 14% in maize, and lowest in faba bean, 17% and 12%, respectively (Tables 1, 2, 3). Thus, hemp was clearly the most difficult raw material for biological treatments. Standard errors for the total carbohydrates (reducing sugars) varied from ± 0.1% to ± 3.1%. Data for total reducing sugars is not shown but standard errors of the monosaccharide results were assumed to remain in the same level. Standard error in lignin analyses remained below ± 0.6%. However, the amount of water-soluble carbohydrates and non-cellulosic glucan content were lowest, about 12% in hemp, as compared with 27% in maize and 35% in faba bean (Tables 1, 2, 3). The hemicellulose and pectin contents were fairly similar in all crops; 16 and 6% ± 0.3% in hemp, 18% and 2% ± 0.1% in maize and 10% and 4% ± 0.1% in faba bean, respectively. The DM content of chopped fresh crops also varied, being 30% in hemp, 24% in faba bean and 17% in maize. The DM content of the hemp harvested in October 2009 was a few percent higher than in September 2008, and reached 63% after 2 days wilting prior to the start of storage. The ash content of the crops varied, being in general about 6% to 8% of the DM (Tables 1, 2, 3). Mass balances of materials and treatments varied from 94.4% to 118.7%. Average for maize was 110.4%, 105.8% for faba bean and 101.4% for hemp. Some acid soluble ash and protein are still present in lignin and are thus overlapping in the mass balance calculations.

**Table 1:** Chemical composition of whole-crop maize before and after ensiling (4 and 8 months) without or with formic acid (0.5% of fresh material)

Components, percentage of dry matter	Fresh	Ensiled without additives		Ensiled with formic acid	
	
	Month 0	Month 4	Month 8	Month 4	Month 8
Dry matter, %	16.7	18.6	17.9	18.8	19.8

WSC^a^	14.0	0.6	8.2	23.9	22.3

Glucose	5.7	0.6	6.5	12.1	11.3

Fructose	8.0	X	X	11.1	10.2

Glucans^b^	13.3	11.0	6.2	11.0	12.1

Cellulose	23.6	26.0	22.4	27.5	24.3

Arabinoxylans	17.0	17.3	16.6	17.8	18.0

Galactans	0.9	0.9	0.6	0.9	0.8

Galacturonic acid	1.7	X	X	X	X

Lignin	14.1	15.8	14.5	15.3	15.8

Protein	10.6	10.6	10.0	10.6	10.6

Ash	8.0	7.9	7.7	7.3	7.5

Samples were extracted with acetone. Water-soluble carbohydrates (WSC) and fructose were determined from the filtrates of the blended crop.

^a^Non-structural carbohydrates expressed as total monosaccharides.

^b^Glucans from non-cellulose parts.

X = < 0.5% of dry matter.

**Table 2:** Chemical composition of fresh hemp before and after ensiling for 4 and 8 months without or with formic acid (1.0% of fresh material) or preserved with urea (3.0% of dry material)

	Harvest year 2008					Harvest year 2009				
	
Components, percentage of dry matter	Fresh	Ensiled without additives		Ensiled with formic acid		Fresh	Ensiled without additives		Preserved with urea	
	
	Month 0	Month 4	Month 8	Month 4	Month 8	Month 0	Month 4	Month 8	Month 4	Month 8
Dry matter, %	30.3	27.8	27.6	28.5	29.3	63.4	58.5	64.5	66.7	68.5

WSC^a^	5.7	1.0	0.9	7.2	6.6	1.7	2.4	1.6	1.3	1.4

Glucose	3.2	0.8	0.7	3.8	3.6	1.0	1.2	0.9	1.0	1.0

Fructose	2.4	X	X	2.1	1.8	0.5	0.5	X	X	X

Glucans^b^	7.5	3.8	3.1	6.4	6.9	2.3	4.8	2.3	4.6	2.2

Cellulose	37.1	41.2	36.3	37.5	40.7	35.9	38.3	42.4	46.5	39.8

Arabinoxylans	12.0	7.8	10.0	9.4	9.7	11.8	8.4	9.3	7.4	9.4

Mannans	2.5	2.7	2.0	2.5	2.4	2.1	2.3	2.4	2.7	1.9

Galactans	1.9	1.1	0.8	1.8	1.7	1.8	1.5	1.5	1.9	1.6

Galacturonic acid	5.7	5.5	5.2	5.3	6.4	4.6	5.0	3.7	5.1	3.9

Lignin	16.9	12.9	16.6	13.4	14.5	16.8	14.0	14.7	13.0	15.0

Protein	7.5	7.5	8.1	8.1	8.1	6.9	6.9	6.3	9.4	10.0

Ash	8.0	9.7	8.2	7.9	7.9	5.0	6.8	6.3	6.6	6.2

Samples were extracted with acetone. Water-soluble carbohydrates (WSC) and fructose were determined from the filtrates of the blended crop.

^a^Non-structural carbohydrates expressed as total monosaccharides.

^b^Glucans from non-cellulose parts.

X = < 0.5% of dry matter.

**Table 3:** Chemical composition of fresh faba bean before and after ensiling for 4 and 8 months without or with formic acid (0.5% of fresh material)

Components, percentage of dry matter	Fresh	Ensiled without additives		Ensiled with formic acid	
	
	Month 0	Month 4	Month 8	Month 4	Month 8
Dry matter, %	24.4	24.7	25.7	25.8	24.8

WSC^a^	5.9	3.3	3.7	8.2	18.3

Glucose	3.6	1.2	1.4	3.6	13.4

Fructose	1.8	X	X	2.0	2.0

Galactose	X	1.2	1.2	1.4	1.5

Arabinose	X	0.8	0.8	1.0	1.2

Glucans^b^	29.1	24.0	28.0	33.4	30.0

Cellulose	16.8	8.5	13.4	15.2	16.9

Arabinoxylan	8.1	8.1	6.5	7.6	7.2

Galactan	1.9	1.0	0.8	1.3	1.1

Galacturonic acid	4.3	3.7	4.2	4.1	3.1

Lignin	12.5	12.6	13.8	13.2	13.2

Protein	18.8	18.8	18.8	19.4	19.4

Ash	7.0	5.9	6.0	5.4	5.1

Samples were extracted with acetone. Water-soluble carbohydrates (WSC) and fructose were determined from the filtrates of the blended crop.

^a^Non-structural carbohydrates expressed as total monosaccharides.

^b^Glucans from non-cellulose parts.

X = < 0.5% of dry matter.

### Ensiling in acidic conditions

Ensiling without any additives was applied as reference for all crops. The pH of the ensiled maize, faba bean and hemp in 2008 decreased as a consequence of microbial acid production, but the addition of 0.5% formic acid to maize and faba bean and 1% to hemp resulted in a more significant and rapid decrease of the pH (Table 4). Ensiling of hemp appeared to suffer from the combination of high pH (7-8) and high DM of the plant material, especially after the 2009 harvest. The pH was not reduced, and only a low amount of lactic acid was formed (Table 4). The low acid production was attributable to the high content of dry matter and low content of WSC. Other indicators of successful ensiling were observed with the other crops, including copious production of lactic and acetic acids, but only trace amounts of butyric, propionic and valeric acids (Table 4). An increase in acetic and lactic acid contents was recorded in the ensiled crops, indicating increased activity of heterofermentative lactic acid bacteria. Noticeable annual differences in oxalic acid concentration in hemp were observed, from only 0.6% of DM in 2008 to almost 5% in 2009. The oxalic acid content was somewhat reduced in the ensiled hemp. When formic acid was added to hemp, no essential changes in the produced acid concentrations were observed.

**Table 4:** The pH, total and ammonium nitrogen, as well as the amounts of acids in fresh and ensiled hemp, maize and faba bean

Crop, year and treatment	Months	pH	NH_3_, g/kg	Percentage of dry matter						
				
				Total N	Oxalic acid	Formic acid	Malic acid	Lactic acid	Acetic acid	VFA
Hemp 2008:										

Fresh	0	8.0	0.6	1.2	0.6	ND	1.3	ND	0.3	ND

No additives	4	5.5	0.6	1.2	ND	ND	1.8	7.8	1.7	0.6
	
	8	6.4	1.8	1.3	ND	ND	2.5	7.3	2.2	0.6

Formic acid	4	4.2	0.6	1.3	1.2	4.3	2.1	ND	0.9	0.4
	
	8	4.3	0.7	1.3	0.8	3.3	1.9	1.4	1.0	0.5

Hemp 2009:										

Fresh	0	7.0	ND	1.1	4.9	ND	0.7	ND	1.2	ND

No additives	4	7.5	1.1	1.1	3.0	ND	2.7	2.2	1.0	0.4
	
	8	8.1	0.9	1.0	2.7	ND	1.2	0.5	0.7	0.4

Urea	4	8.6	14.3	1.5	5.7	ND	0.8	ND	2.1	0.4
	
	8	8.7	8.4	1.6	5.1	ND	0.9	ND	2.1	0.4

Maize 2008:										

Fresh	0	5.5	0.3	1.7	9.2	ND	2.0	ND	ND	1.0

No additives	4	4.3	0.7	1.7	9.6	0.9	0.7	8.2	3.0	1.2
	
	8	4.2	0.8	1.6	9.0	0.9	0.8	7.5	2.9	1.2

Formic acid	4	3.7	0.1	1.7	9.9	3.6	2.1	ND	ND	1.6
	
	8	3.7	ND	1.7	6.8	2.4	1.4	ND	ND	0.4

Faba bean 2008:										

Fresh	0	5.6	ND	3.0	0.1	ND	2.3	ND	0.7	1.1

No additives	4	4.6	0.8	3.0	0.1	ND	2.5	8.2	2.3	0.5
	
	8	4.6	1.4	3.0	0.1	ND	2.7	8.8	2.3	0.5

Formic acid	4	4.2	0.5	3.1	0.2	3.7	3.2	2.1	0.9	0.7
	
	8	4.1	1.4	3.1	0.4	4.0	3.6	2.7	1.0	0.6

Acids and NH3 were determined from the filtrates of the blended crops.

^a^Prewilted for urea ensiling.

^b^Expressed as % of dry matter.

ND = not detected; VFA = volatile fatty acids (propionic, butyric, valeric and isovaleric).

**Figure 1:**
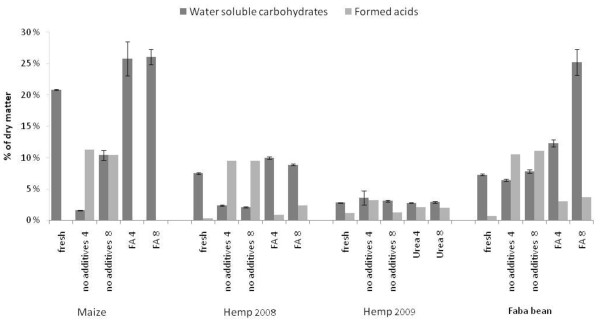
**Water-soluble carbohydrates, expressed as total reducing sugars, and acids formed during the storing of various crops (percentage of dry matter)**.

The WSC in maize and hemp consisted mainly of glucose and fructose (Tables 1 and 2), and decreased significantly during ensiling without formic acid. Most of the WSC in hemp and maize was consumed already during the first 4 months of ensiling, whereas in faba bean, only about half of the WSC was consumed. Prolonged storage appeared to liberate more sugars into this fraction from the easily hydrolysable polymers, as the WSC content increased between 4 and 8 months of ensiling without additives. In all cases, the WSC content increased in the formic acid-preserved materials. The higher the amount of formed acids, the lower was the residual WSC in the sample (Table 3 and Figure 1). When formic acid was supplied, the formation of fermented acids, namely lactic and acetic acids, was reduced, and the soluble carbohydrates were well preserved. The very low content of WSC in the dry hemp material in 2009 correlated well with the low lactic acid content in the ensiled hemp, indicating unfavourable growth conditions for lactic acid bacteria. In faba bean (Table 3), the concentration of WSC decreased slightly in the ensiled samples, whereas in samples treated with formic acid, the amount was significantly increased, probably due to hydrolysis of starch.

Minor changes in the amounts of structural carbohydrates occurred during ensiling. The amount of the insoluble residue (Klason lignin and ash) decreased by 3% to 4% in hemp where the original concentration was also highest (Table 2) but no decrease in either maize or faba bean lignin could be observed (Tables 1 and 3). Changes in galacturonic acid content, originating from pectin, during ensiling were not considerable. Indeed, the change in the content of total carbohydrates was mainly explained by the change in the amount of water-soluble carbohydrates. The cellulose content remained almost constant, and in the preserved materials, only modest enrichment or decrease in carbohydrate contents occurred. Only the hemicellulose fraction of faba bean was partially hydrolysed, as indicated by the increase of galactose and arabinose in the WSC fraction (Table 3).

### Alkaline preservation

Alkaline treatment was successfully applied to hemp (Table 4), which had a sufficiently high DM content to allow alkaline preservation. Preliminary inspection showed that the two urea concentrations gave very similar results in terms of composition of the material and evaluation for bioconversions. Thus, only the results of treatments with the lower urea dosage (3% w/w) are presented here. Preserving hemp with urea retained the water-soluble carbohydrates at a similar low level as detected in the fresh material (Table 2), increased the pH to almost 9.0 and, as expected, significantly enhanced the total and ammonium-derived amounts of nitrogen. The share of ammonium nitrogen increased from less than 0.1% up to 1.4% of DM (Table 4) and the total nitrogen content increased by half, leading to an apparent increase of crude protein content from 6.5% to 9.7% of DM (Table 2). The carbon to nitrogen ratio was consequently decreased from 43 to 28. The high oxalic acid content was retained during the alkaline preservation. The total material loss was very low, and only minor changes in the amounts of structural polymers were observed with the analytical systems used in this study (Table 2). The determined values remained practically unaltered, indicating good preservation of the biomass during alkaline preservation.

### Potential of preserved crops for methane production

**Figure 2:**
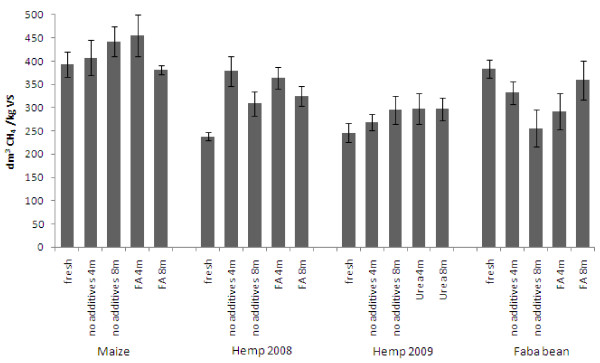
**Specific methane yields from the studied crops**. Yields are expressed as volume of methane per weight of corrected volatile solids.

Production of methane from urea-treated hemp increased by 25% to 42% compared to the yield from the fresh material, and even higher yields were found after acid ensiling in 2008. Hemp ensiled for 4 months without additives produced over 50% higher methane yields than the fresh hemp (Figure 2). The methane yields from hemp were 309-379 dm^3 ^CH_4_/kg VS. After 8 months, the increase was slightly lower, especially without formic acid addition. The methane yield of fresh hemp in 2009 was similar to that of the previous year, although it was harvested 1 month later. While ensiling was particularly important for efficient methane production from hemp, it also resulted in substantially increased methane yields from maize, exceeding that obtained from fresh material (Figure 2). Use of formic acid in ensiling of maize enhanced the methane yields even further, increasing after 4 months ensiling to 455 dm^3 ^CH_4_/kg VS, that is, by 16% from 393 dm^3 ^CH_4_/kg VS, obtained from the fresh material. Ensiled faba bean, in contrast, yielded somewhat less methane than the fresh material.

### Potential of preserved crops for enzymatic conversion to sugars

**Figure 3:**
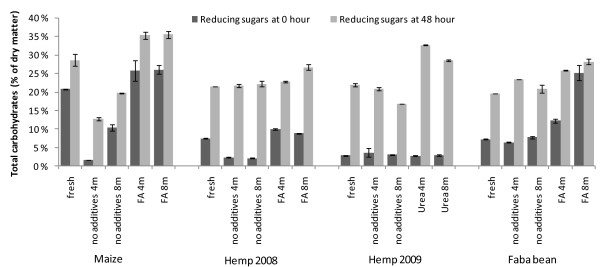
**Enzymatic hydrolysis of the studied substrates expressed as reducing sugars (percentage of dry matter)**. **(a) **Maize, **(b) **faba bean, **(c) **hemp harvested in 2008 and **(d) **hemp harvested in 2009.

**Figure 4:**
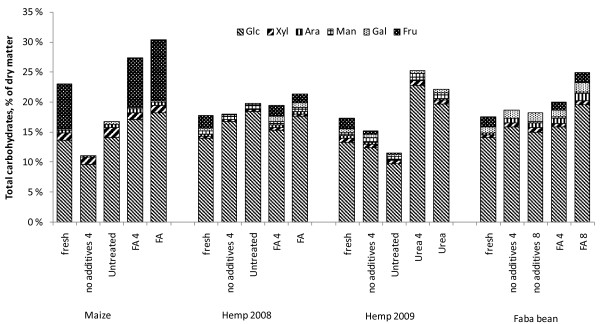
**Composition of the studied substrates after enzymatic hydrolysis, expressed as monosaccharides (percentage of dry matter)**. Sugars determined by high performance anion exchange chromatography with pulsed amperometric detection (HPAEC-PAD).

Urea-based preservation was the most successful method for increasing the enzymatic hydrolysis of hemp (Figures 3 and 4). Preservation of hemp with urea for 4 months resulted in the best carbohydrate yields and total conversion in enzymatic hydrolysis tests of the stored material, that is, 33% reducing sugars of the dry matter. The main increase originated from glucose, although an increase in galactose content was also observed. The difference between reducing sugars and total identified monosaccharides of fresh and preserved hemp remained at the same level, 23% to 26%, which indicates that the share of oligosaccharides in the hydrolysates of fresh and preserved hemp was about the same. In contrast, the share of oligosaccharides in the formic acid-ensiled hemp was lower, the difference between reducing sugars and total monosaccharides being only 11% to 20%. The difference between the monosaccharides and reducing sugars is due to the oligosaccharides not being hydrolysed with the enzyme preparations used. The alkaline and acid preservation conditions may result in different oligomer patterns due to hydrolysis of, for example, acetyl substituents or other alkaline labelled carbohydrate residues. The main oligomers present in the raw materials before hydrolysis were long xylo-oligomers (data not shown).

The hemp harvested in 2009 with the higher dry matter content and further prewilted material contained considerably less WSC than the sample of 2008. The conversion of polymers was about the same and ensiling without additives did not increase the carbohydrate yield (Figure 3). Fresh samples of the 2008 hemp crop, ensiled without and with formic acid, all produced similar results in the hydrolysis tests, although some increment of released sugars, mostly of glucose, was observed in formic acid ensiled samples after prolonged storage times (Figure 4).

The total carbohydrate yield from the fresh material was highest in maize, 29% of DM (Figure 3), mostly due to the water-soluble carbohydrates. The amount of total carbohydrates from formic acid-ensiled maize increased up to 36% of dry matter (Figure 3). However, this was due to the increased WSC released during the ensiling process, and the enzymatic conversion of actual polymers remained the same. The conversion of the WSC to acids during ensiling without formic acid strongly decreased the total amount of easily available carbohydrates after enzymatic hydrolysis. Glucose and fructose were the only monosaccharides increased after the enzymatic hydrolysis after acidic ensiling (Figure 4).

The accessibility to enzymatic hydrolysis of faba bean increased only slightly in the ensiled and acid-ensiled samples compared to the fresh material (Figure 3). As with the other raw materials, the positive effect of ensiling was observed as preservation of the soluble carbohydrates, adding to the overall yield of soluble carbohydrates after enzymatic hydrolysis. Besides glucose, minor increases in content of arabinose and galactose were observed (Table 3).

## Discussion

### Ensiling and chemical composition

The three species selected for the work are potential crops to be used as raw materials for biofuel production, as their cultivation and management are well adapted for boreal conditions [22]. Alkaline preservation and ensiling proved to be suitable methods to store the biomass, thus allowing its use for energy throughout the year. The pH, low amounts of volatile fatty acids formed and the limited degradation of protein in ensiled crops indicated that the ensiling process succeeded well in all three.

Formation of lactic acid from the readily available carbohydrates by natural microflora is the main acidifying agent preserving the non-treated raw material. Additives, such as acids or bases, are used to accelerate the pH change in order to decrease or prevent the loss of carbohydrates and formation of other acids. The benefits of this process were clearly seen in this work. In samples ensiled without additives, the content of water-soluble carbohydrates decreased as acids were formed during the fermentation, while addition of formic acid resulted in increased amounts of WSC (consisting mainly of glucose and fructose). Part of the increase in glucose and fructose is possibly due to degradation of sucrose present in the herbaceous crops [2]. In crops ensiled with formic acid, some sugars were obviously converted to lactate and acetate, and the further increment of WSC was attributable to the further hydrolysis of the easily hydrolysable components of the biomass. Especially in faba bean, arabinose and galactose were converted into the water-soluble fraction during the ensiling process. Pectins, remaining at similar levels in faba bean and hemp, appeared either not to be used as carbon source or to be more recalcitrant inside the fibrous structures. However, a minor decrease in pectin was detected in alkaline-treated hemp.

Some lignin was lost during alkaline preservation and ensiling of hemp, which could indicate loosening of the rigid chemical structure. Nevertheless, lignin degradation is considered a strictly aerobic process, and although some simple phenolic compounds may be degraded by anaerobic bacteria [30], degradation of lignin is not expected to take place during the ensiling conditions. Alterations of other components such as protein may have influenced the measured lignin content. Similarly, neutral detergent fibre content measurement was interfered by the released structural nitrogen during ensiling [31]. However, the lignin fraction in urea preserved is clearly lower compared to the fresh hemp if all the protein is subtracted from the acid insoluble residue after acid hydrolysis. This may indicate the real decrease of lignin.

Ensiling aims at preventing spoilage and minimising the loss of material. Losses include conversion of carbohydrates to CO_2 _and to undesired fermentation products. Although the dry matter loss of hemp was not measured after ensiling in 2008, the loss in 2009 was less than 1%. Nevertheless, losses of energy are often lower than the losses in DM, since the formed fermentation products during ensiling have a higher gross energy (GE) value than the original substrates [2]. Different additives and successful anaerobic ensiling process clearly have a strong impact on the material loss and conversion of one compound to another.

### Biogas production

The formed acids in the ensiled material obviously contributed to the subsequent biogas yield, as bacteria in the inoculum first hydrolyse the carbohydrate polymers into sugars that are further fermented to carboxylic acids and finally to carbon dioxide, hydrogen and acetate, the intermediates of methane production. However, the excessive formation of acids in the digester may decrease the pH and inhibit methanogens, thus causing a further accumulation of the acids as intermediate products of the digestion process. In practise, a sufficient buffering capacity (alkalinity) of the process helps to keep the pH close to neutral and avoid the digester upset. In the present biogasification tests, the acid concentrations were low enough and pH remained neutral. Addition of formic acid appears to be an attractive option as a preservation agent for biogas production, resulting in loosening of the plant cell wall structure along with good preservation of WSC, thus maximising the methane yield after the ensiling. The cause of the decrease in methane production in formic acid-treated maize after prolonged storage is not clear, because the chemical composition of the material ensiled for 4 and 8 months was similar. Alkaline preservation provided only a minor increase in methane production from hemp, although the enzymatic hydrolysability was enhanced. The high dry matter content may, however, have restricted acid formation and indeed, drying has not been found to be a beneficial storing method prior to methane production [32].

Ensiling of maize without additives has been shown to be suitable for biogas production [15,33]. Although maize with its high WSC content was an excellent fresh raw material [22], ensiling improved its methane production further and an additional increase was obtained when formic acid was used. In this study, fresh maize was found to be superior to hemp and faba bean for biogas production, whereas hemp reached the same methane yields as maize after ensiling.

Ensiling did not improve the potential of faba bean as a raw material for methane production. Methane yields from all ensiled faba bean samples were significantly lower than those of the fresh crop. The only exception was the one ensiled with acid for 8 months having no significance effect compared to the fresh material. Faba bean is the most protein-rich and starch-rich of the three studied crops. Protein is a better substrate for methane production than carbohydrates [17] and thus, this crop may not benefit from loosening the structure during ensiling as much as the cellulose-rich hemp. Starch in the fresh beans is more readily available than carbohydrates from structural polymers in the plant cell walls. The use of faba bean or other grain legumes as energy crops could become more attractive in crop mixtures in short-season zones where its rapid growth is important, or in contexts where the beans would be used for food or feed, and the residue for energy production.

### Enzymatic hydrolysability

Although acidic ensiling had a positive effect on biogas production of hemp, it did not enhance its accessibility to enzymatic hydrolysis as clearly. In contrast, alkaline preservation significantly enhanced the availability of carbohydrates to the enzymatic conversion of this crop. In maize, the high original amount of WSC in the fresh material contributed 14% of the dry matter, comprising up to 49% of the carbohydrate yield after enzymatic hydrolysis. Therefore, addition of formic acid was particularly important while ensiling maize, as the valuable WSC fraction was well preserved. The WSC in faba bean increased as well after ensiling with formic acid, resulting in total carbohydrates of about 30% of DM. The commercial enzyme preparation Celluclast is not specifically optimised for the hydrolysis of fresh agricultural crops, containing various hemicelluloses, pectins and soluble oligomers. Thus, some of the water-soluble carbohydrates, formed during the ensiling, may not have been hydrolysed further. Generally, the amount of sugars liberated in the enzymatic hydrolysis of all crops remained low, which indicates that the lignocellulose structure was not extensively loosened during the mild conditions of ensiling. The structure and composition of the three crops may cause different limitations to the enzymatic hydrolysis using standard enzyme preparation and dosage. Therefore, the aim was rather to compare the effects of storing methods than to evaluate or optimise the hydrolysis of various crops. Addition or formation of organic acids may interfere with the enzymatic hydrolysis [34], although the concentration of formed lactic and acetic acids remained low. Thus, the hydrolytic efficiency was not assumed to be affected by the formed acids. However, lactic, acetic and formic acids have been used to catalyse the thermal pretreatments of corn stover prior to hydrolysis into fermentable sugars. Although lactic acid has been shown to slightly inhibit the enzymatic hydrolysis, all three treatments improved the enzymatic conversion [35,36].

The present results demonstrated that anaerobic storing is a suitable storage method for annual crops, preserving carbohydrates and even enhancing the enzymatic hydrolysis to platform sugars. Anaerobic preservation may be introduced as a decentralised mild pre-pretreatment method at the farm sites prior to more energy consuming treatments. Additionally, biogas production from the residue of enzymatic hydrolysis has been studied in order to further improve the total energy balance of the process [37]. The data presented in this work clearly shows the importance of the choice of the storage method for various crop raw materials, potentially affecting the conversion efficiency to various biofuel applications.

## Conclusions

Ensiling with formic acid was a particularly efficient method for storing maize and faba bean, and the yields of total fermentable carbohydrates in these species were fully retained or even increased during the storage. Ensiling of maize and hemp proved to be viable options for biogas production, as the biogas yields were remarkably increased in all treatments studied. Drier hemp was stored additionally in alkaline conditions that increased the accessibility of the raw material for enzymatic hydrolysis even more than ensiling with formic acid. While all studied crops still require a more powerful pretreatment to release the maximum amount of carbohydrates, ensiling is indeed a suitable decentralised storing and pretreatment method prior to further processing to platform sugars or energy carriers.

## Competing interests

The authors declare that they have no competing interests.

## Authors' contributions

AP planned and coordinated the experiments, carried out biogas potential experiments and hydrolysis studies, analysed the results, and drafted the manuscript. PM contributed to the design of the study and analysis of the results, FLS provided the plant materials and the plant science expertise, SJ provided the ensiling facilities and expertise, and MK provided the biogas conversion facilities and expertise. LV conceived and coordinated the overall study, and helped to analyse the results and finalise the paper. All authors contributed to the preparation and finalisation of the manuscript, and FLS checked the language.

## References

[B1] Dewar WA McDonald P Whittenbury R The hydrolysis of grass hemicelluloses during ensilage J Sci Food Agric 1963 14 411 417 10.1002/jsfa.2740140610

[B2] McDonald P Henderson AR Heron SJE The Biochemistry of Silage 1991 Aberytstwyth, UK: Cambrian Printers Ltd

[B3] Lehtomäki A Biogas production from energy crops and and crop residues PhD thesis 2006 Biological and Environmental Science Department, University of Jyväskylä

[B4] Guedes CM Rodriques MM Gomes MJ Silva SR Ferreira LM Mascarenhas-Ferreira A Urea treatment of whole-crop triticale at four growth stages: effects on chemical composition and on in vitro digestibility of cell wall J Sci Food Agric 2006 86 964 970 10.1002/jsfa.2444

[B5] Jaakkola S Kaunisto V Huhtanen P Volatile fatty acid proportions and microbial protein synthesis in the rumen of cattle receiving grass silage ensiled with different rates of formic acid Grass Forage Sci 2006 61 282 292 10.1111/j.1365-2494.2006.00532.x

[B6] Hendricks ATWM Zeeman G Pretreatments to enhance the digestibility of lignocellulosic biomass Bioresour Technol 2009 100 10 18 10.1016/j.biortech.2008.05.027 18599291

[B7] Mosier N Wyman C Dale B Elander R Lee Y Holtzappe M Ladisch M Features of promising technologies for pretreatment of lignocellulosic biomass Bioresour Technol 2005 96 673 686 10.1016/j.biortech.2004.06.025 15588770

[B8] Kumar P Barret DM Delwiche M Stroeve P Methods for pretreatment of lignocellulosic biomass for efficient hydrolysis and biofuel production Ind Eng Chem Res 2009 48 3713 3729 10.1021/ie801542g

[B9] Digman MF Shinners KJ Casler MD Dien BS Hatfield RD Jung HG Muck RE Weimer PJ Optimizing on-farm pretreatment of perennial grasses for fuel ethanol production Bioresour Technol 2010 101 5305 5314 10.1016/j.biortech.2010.02.014 20202834

[B10] Huber JT Thomas JW Urea-treated corn silage in low protein rations for lactating cows J Dairy Sci 1970 54 224 230

[B11] Digman MF Shinners KJ Muck RE Dien BS Pilot-scale on-farm pretreatment of perennial grasses with dilute acid and alkali for fuel ethanol production Transact ASABE 2010 53 1007 1014 10.1016/j.biortech.2010.02.014 20202834

[B12] Kim TH Lee YY Pretreatment and fractionation of corn stover by ammonia recycle percolation process J Biotech 2005 96 2007 2013 10.1016/j.biortech.2005.01.015 16112488

[B13] Bals B Murnen H Allen M Dale B Ammonia fiber expansion (AFEX) treatment of eleven different forages: Improvements to fiber digestibility *in vitro* Anim Feed Sci Tech 2010 155 2 4

[B14] Sun Y Cheng J Hydrolysis of lignocellulosic materials for ethanol production: a review Bioresour Technol 2002 83 1 11 10.1016/S0960-8524(01)00212-7 12058826

[B15] Amon T Amon B Kyvoruchko V Zollitsch W Mayer K Gruber L Biogas production from maize and dairy cattle manure - influence of biomass composition on the methane yield Agric Ecosyst Environ 2007 118 173 182 10.1016/j.agee.2006.05.007

[B16] Neureiter M dos Santos JTP Lopez CP Pichler H Kirchmayr R Braun R Ahring BK, Hartmann H Effect of silage preparation on methane yields from whole crop maize silages In Proceedings of the 4th International Symposium on Anaerobic Digestion of Solid Waste: 31 August-2 September 2005; Water Sci Techn 2005 53 Copenhagen, Denmark 109 115

[B17] Pakarinen O Lehtomäki AM Rissanen S Rintala J Storing energy crops for methane production: effects of solids content and biological additive Bioresour Technol 2008 99 7074 7082 10.1016/j.biortech.2008.01.007 18328694

[B18] Vervaeren H Hostyn K Ghekiere G Willems B Biological ensilage additives as pretreatment for maize to increase the biogas production Renew Energ 2010 35 2089 2093 10.1016/j.renene.2010.02.010

[B19] Murphy PT Moore KJ Richard TL Bern CJ Enzyme enhanced solid-state fermentation of kenaf core fiber for storage and pretreatment Bioresour Technol 2007 98 3106 3111 10.1016/j.biortech.2006.10.032 17222553

[B20] Chen Y Sharma-Shivappa RR Chen C Ensiling agricultural residues for bioethanol production Appl Biochem Biotechnol 2007 143 80 92 10.1007/s12010-007-0030-7 18025598

[B21] Thomsen MH Holm-Nielsen JB Oleskowicz P Thomsen AB Pretreatment of whole-crop harvested, ensiled maize for ethanol production Appl Biochem Biotechnol 2008 148 23 33 10.1007/s12010-008-8134-2 18418738

[B22] Pakarinen A Maijala P Stoddard F Santanen A Kymalainen M Viikari L Evaluation of annual bioenergy crops in the Boreal zone for biogas and ethanol production Biomass Bioenergy 2011 35 3071 3078 10.1016/j.biombioe.2011.04.022

[B23] Faulkner JS A comparison of faba beans and peas as whole crop forages Grass Forage Sci 1985 40 161 169 10.1111/j.1365-2494.1985.tb01733.x

[B24] Tetlow MR Stark BA, Wilkinson JM A decade of research into whole-crop cereals at Hurley Whole-Crop Cereals 1992 Southampton, UK: Chalcombe Publications 1 19

[B25] Sluiter A Hamnes B Ruiz R Scarlata C Sluiter J Templeton D Crocker D Determination of structural carbohydrates and lignin in biomass. Laboratory analytical procedure http://www.nrel.gov/biomass/analytical_procedures.html

[B26] Miller GL Use of dinitrosalicylic acid reagent for determination of reducing sugars Anal Chem 1959 31 426 428 10.1021/ac60147a030

[B27] Sundberg A Sundberg K Lillandt C Holmbom B Determination of hemicelluloses and pectins in wood and pulp fibres by acid methanolysis and gas chromatography Nord Pulp Pap Res J 1996 11 216 219 10.3183/NPPRJ-1996-11-04-p216-219

[B28] Weischbach F Strubelt C Correcting the dry matter content of maize silages as a substrate for biogas production Landtechnik 2008 2 82 83

[B29] Huida L Väätäinen H Lampila M Comparison of dry matter contents in grass silages as determined by oven drying and gas chromatographic water analysis Annales Agriculturae Fenniae 1986 25 215 230

[B30] Schink B Phillipp B Muller J Anaerobic degradation of phenolic compounds Naturwissenchaften 2000 87 12 23 10.1007/s001140050002 10663127

[B31] Rinne M Jaakkola S Huhtanen P Grass maturity effects on cattle fed silage-based diets. 1. Organic matter digestion, rumen fermentation and nitrogen utilization Anim Feed Sci Tech 1997 67 1 17 10.1016/S0377-8401(96)01141-8

[B32] Egg R Coble C Engler C Lewis D Feedstock storage, handling and processing Biomass Bioenergy 1993 5 71 94 10.1016/0961-9534(93)90009-S

[B33] Plöchl M Zacharias H Herrmann C Heiermann M Prochnow A Influence of silage additives on methane yield and economic performance of selected feedstock Agric Eng Int 2009 11 1123

[B34] Walton S Van Heiningen A Van Walsum P Inhibition effects on fermentation of hardwood extracted hemicelluloses by acetic acid and sodium Bioresour Technol 2010 101 1935 1940 10.1016/j.biortech.2009.10.043 19944597

[B35] Xu J Thomsen MH Thomsen AB Enzymatic hydrolysis and fermentability of corn stover pretreated by lactic acid and/or acetic acid J Biotechnol 2009 139 300 305 10.1016/j.jbiotec.2008.12.017 19297725

[B36] Xu J Thomsen MH Thomsen AB Pretreatment on corn stover with low concentration of formic acid J Microbiol Biotechnol 2009 19 845 850 19734724

[B37] Kaparaju P Serrano M Thomsen AB Kongjan P Angelidaki I Bioethanol, biohydrogen and biogas production from wheat straw in a biorefinery concept Bioresour Technol 2009 100 2562 2568 10.1016/j.biortech.2008.11.011 19135361

